# Baseline tebuconazole sensitivity and potential resistant risk in *Fusarium graminearum*

**DOI:** 10.1186/s12870-024-05206-1

**Published:** 2024-08-21

**Authors:** Feng Zhou, Xiaoli Zhou, Yan Jiao, Aohui Han, Huanhuan Zhou, Zeyuan Chen, Weiguo Li, Runqiang Liu

**Affiliations:** 1 Henan Engineering Research Center of Green Pesticide Creation and Pesticide Residue Monitoring by Intelligent Sensor, Henan Institute of Science and Technology, Xinxiang, 453003 China; 2 Postdoctoral Research Base, Henan Institute of Science and Technology, Xinxiang, 453003 China; 3 School of Food Science and Engineering, Henan University of Technology, Zhengzhou, 450001 China; 4 Henan Engineering Research Center of Crop Genome Editing / Henan International Joint Laboratory of Plant Genetic Improvement and Soil Remediation, Henan Institute of Science and Technology, Xinxiang, 453003 China

**Keywords:** *Fusarium graminearum*, Tebuconazole, Baseline sensitivity, Cross-resistance, Resistant mechanism

## Abstract

**Background:**

The *Fusarium* head blight caused by *Fusarium graminearum* results in reduced crop yields and the potential for vomitoxin contamination, which poses a risk to both human and livestock health. The primary method of control relies on the application of chemical fungicides.

**Results:**

The current study found that the tebuconazole sensitivity of 165 *F. graminearum* isolates collected from the Huang-Huai-Hai region of China between 2019 and 2023 ranged from 0.005 to 2.029 µg/mL, with an average EC_50_ value of 0.33 ± 0.03 µg/mL. The frequency distribution conformed to a unimodal curve around the mean, and therefore provides a useful reference for monitoring the emergence of tebuconazole resistance in field populations of *F. graminearum*. No cross-resistance was detected between tebuconazole and other unrelated fungicides such as flutriafol, propiconazole and fluazinam, but there was a clear negative cross-resistance with triazole fungicides including fludioxonil, epoxiconazole, hexaconazole, and metconazole. Analysis of five tebuconazole-resistant mutants produced under laboratory conditions indicated that although the mycelial growth of the mutants were significantly (*p* < 0.05) reduced, spore production and germination rates could be significantly (*p* < 0.05) increased. However, pathogenicity tests confirmed a severe fitness cost associated with tebuconazole resistance, as all of the mutants completely loss the ability to infect host tissue. Furthermore, in general the resistant mutants were found to have increased sensitivity to abiotic stress, such as ionic and osmotic stress, though not to Congo red and oxidative stress, to which they were more tolerant. Meanwhile, molecular analysis identified several point mutations in the *CYP51* genes of the mutants, which resulted in two substitutions (I281T, and T314A) in the predicted sequence of the *FgCYP51A* subunit, as well as seven (S195F, Q332V, V333L, L334G, M399T, E507G, and E267G) in the *FgCYP51C* subunit. In addition, it was also noted that the expression of the *CYP51* genes in one of the mutants, which lacked point mutations, was significantly up-regulated in response to tebuconazole treatment.

**Conclusions:**

These results provide useful data that allow for more rational use of tebuconazole in the control of *F. graminearum*, as well as for more effective monitoring of fungicide resistance in the field.

**Supplementary Information:**

The online version contains supplementary material available at 10.1186/s12870-024-05206-1.

## Background

Wheat is one of the most important of all food crops, and is widely planted all over the world, with the area under cultivation and yield of China being among the highest of any nation [1, 2]. Indeed, the Huang-Huai-Hai plain, the main wheat producing region of China [3], alone covers an area of 2305 thousand Ha and accounts for 1.4 million tons, with an average yield of 5.8 tons per Ha, which represented 20% of total grain production in 2023 according to the National Bureau of Statistics of the people’s Republic of China (http://www.stats.gov.cn/sj/zxfb/202307/t20230715_1941239.html). Wheat is primarily a source of dietary calories as a result of its high starch content, but is also a source of fats, protein, vitamins and other nutrients [4], and aside from it use in staple foods such as bread and pasta, it is also an important component of processed foods including the fermentation of bear and edible fungi [5, 6], as well as in the production of livestock feed. Therefore ensuring the safe production of wheat plays an important role in the food security of China, and maintaining quality of life.

*Fusarium* head blight is a common epidemic disease that affects most wheat growing regions of the world. In china the disease is primarily caused by *Fusarium graminearu*m and *Fusarium pseudograminearum* [7], with the primary source of infection being the ascospores produced by fungal colonies growing saprophytically on the remains of dead host tissue. Under suitable climatic conditions, when the temperature is warm and the humidity is high, infection can spread quickly throughout the crop causing symptoms of stem rot, seedling blight, and spike rot [8]. *Fusarium* head blight not only has the potential to seriously reduce yields, but can also affect the quality of the grains [9, 10], as *F. graminearum* produces toxins [11] such as Deoxynivalenol (DON) and Zearalenone (ZEN), which pose a threat to the health of both humans and livestock [12]. Given the wide distribution, rapid disease progression, and potential for substantial economic loss, it is not surprising that the chemicals used for the prevention and control of this devastating disease have frequently been misused resulting in *F. graminearum* developing resistance to a variety of fungicides including carbendazim [13, 14].

Tebuconazole is a triazole fungicide developed by the Bayer Group in Germany, and put into production in 1986. Tebuconazole has high antifungal activity that can not only reduce the growth of *F. graminearum*, but also prevent the accumulation of toxins in the wheat grains of infected plants [15]. In China, tebuconazole has been registered for the control of *Fusarium* head blight since 2009 (http://w.icama.cn/zwb/dataCenter?hash=reg-info, 2023, October, 24). However, a recent survey of *F. graminearum* isolates collected in the Henan Province of China revealed that as a many as 32.4% of field isolates could be considered resistant to tebuconazole when using a discriminatory dose of 5 µg/mL, with the resistant isolates having EC_50_ values that ranged from 0.02 ~ 3.31 µg/mL, while the remaining 67.6% of isolates were still sensitive to tebuconazole with an average EC_50_ of just 0.01 µg/mL [16]. These results were similar to those of Anderson et al., who assessed field isolates of *F. graminearum* in the USA [17] and found that there was also a degree of cross-resistance between tebuconazole and metconazole. Likewise, the study of Zhang et al. found that some metconazole-resistant laboratory mutants exhibited cross-resistance with tebuconazole and the imidazole DMI fungicide prochloraz, but interestingly not with the triazole fungicide prothioconazole [18]. Meanwhile, Ye et al. found no evidence of cross-resistance between tebuconazole and non-triazole fungicides with diverse modes of action, and in addition that tebuconazole-resistance was associated with fitness costs such as reduced mycelial growth [19, 20].

Previous research has shown that the resistance mechanism of triazole fungicides is associated with polygenes, and it is widely believed that the *CYP51* protein from the ergosterol biosynthesis pathway is the main target site of triazole fungicides, and also the main source of resistance as a result of amino acid changes to its primary structure, or changes in its level of expression. The *CYP51* protein in *Fusarium* species occurs as three distinct subunits *FgCYP51A*, *FgCYP51B* and *FgCYP51C* [21], and a recent study found that tebuconazole resistance in *F. graminearum*, might be associated with the up-regulation of *FgCYP51A* expression [18]. However, an earlier study of the DMI fungicide prochloraz found a similar up-regulation of the *FgCYP51B* gene, and that resistance was also associated with reduced fungicide affinity on account of the Y123H mutation in the primary structure of the *FgCYP51B* protein [22]. Meanwhile, molecular analysis of the three *F. graminearum CYP51* subunits in transgenic yeast, found that only *FgCYP51A* and *FgCYP51B* could rescue mutants lacking a functional *CYP51*, which might indicate that Fg*CYP51*C has an unrelated function [23]. Meanwhile, another study that assessed *F. graminearum* deletion mutants found that the absence of *CYP51*A dramatically increased sensitivity to seven DMI fungicides, whilst the loss of *CYP51*B had no effect, and that the loss of *CYP51*C increased sensitivity to some of the DMI fungicides, but not to others [24]. Despite these discoveries, further research is still required to provide a more complete understanding of tebuconazole resistance in *F. graminearum*, and the mechanisms that might cause it. The objectives of the current study were therefore to establish a reliable baseline tebuconazole sensitivity for wild-type isolates collected in the field, assess the biological characteristics of tebuconazole-resistant laboratory mutants, and investigate possible resistance mechanisms using expression analysis and gene sequencing.

## Results

### Tebuconazole sensitivity of 165 *F. Graminearum* isolates collected in Huang-Huai-Hai, China baseline sensitivity

**Fig. 1:**
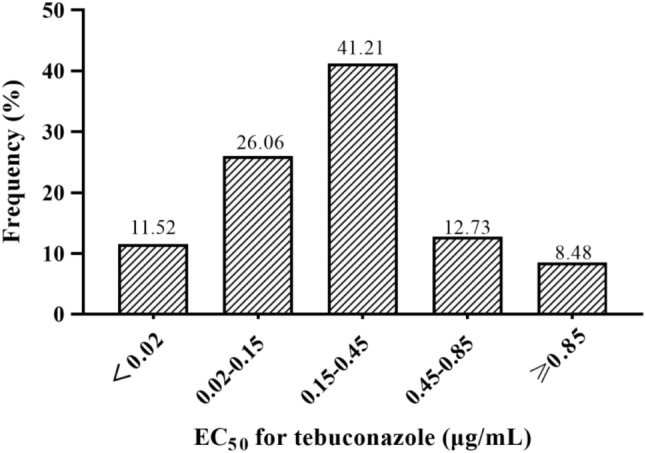
Frequency distribution of tebuconazole EC_50_ values from 165 *F. graminearum* collected in Huang-Huai-Hai, China between 2019 and 2023

Mycelial growth assays using PDA amended with a range of tebuconazole concentrations were used to determine the EC_50_ of 165 field isolates of *F. graminearum*, which were found to have values ranging from 0.005 to 2.029 µg/mL (Supplementary Table 1), with an average EC_50_ of 0.33 ± 0.03 µg/mL. The frequency distribution produced a unimodal curve with most isolates having an EC_50_ in the range of 0.15 ~ 0.45 µg/mL (Fig. 1). Taken together, these results indicate that the average EC_50_ of 0.33 was a suitable baseline sensitivity for monitoring the emergence of tebuconazole resistance in the Huang-Huai-Hai region of China. It was also noted that although the normal distribution of the frequency data indicated that fungicide resistance was not yet widespread in the region, the identification of one isolate with an EC_50_ of 2.029 µg/mL, which was almost an order of magnitude greater than the baseline, could be an early indicator that tebuconazole resistance might be emerging in the wheat fields of Huang-Huai-Hai.

### Change in sensitivity between 2019 and 2023

Although there were significant (*p* < 0.05) differences between the average EC_50_ values of the isolates collected in the five years of the study, there was no particular trend, with the average EC_50_ dropping in the second year, and then again in the third year, before increasing in the fourth, and then again dropping in the fifth (Table 1). The data also revealed a high degree of variation in the EC_50_ values among the isolates collected within the same year, with the greatest variation occurring in 2020 (difference multiple of 146.7), and the lowest in 2021 (difference multiple of 2.4). Taken together, these results provide further evidence that the emergence of tebuconazole resistance, if any, was in its early stages, since although there was some evidence of a putative resistant isolate being collected in 2022 (EC_50_ of 2.029 µg/mL), there was no general trend of increased average EC_50_, or increased variation in EC_50_, which might indicate increasing incidence of tebuconazole resistance over the duration of the study.

**Table 1:** Tebuconazole sensitivity of 165 *F. graminearum* collected in Huang-Huai-Hai, China between 2019 and 2023

Years	Number of isolates (from separate plants)	Range of EC_50_ values	Difference multiple	X ± SD
2019	38	0.044∽0.481	11.0	0.28 ± 0.019 b
2020	32	0.005∽0.757	146.7	0.11 ± 0.029 bc
2021	11	0.013∽0.031	2.4	0.02 ± 0.002 c
2022	40	0.021∽2.029	96.6	0.66 ± 0.092 a
2023	44	0.059∽0.876	14.8	0.31 ± 0.03 b
Total	165	0.005∽2.029	393.4	0.33 ± 0.029 b

*Note* Different letters within a column indicate significant differences according to Fisher’s least test (*p* < 0.05)

### Biological characteristics of four tebuconazole-resistant mutants of *F. Graminearum* Cross-resistance

**Fig. 2:**
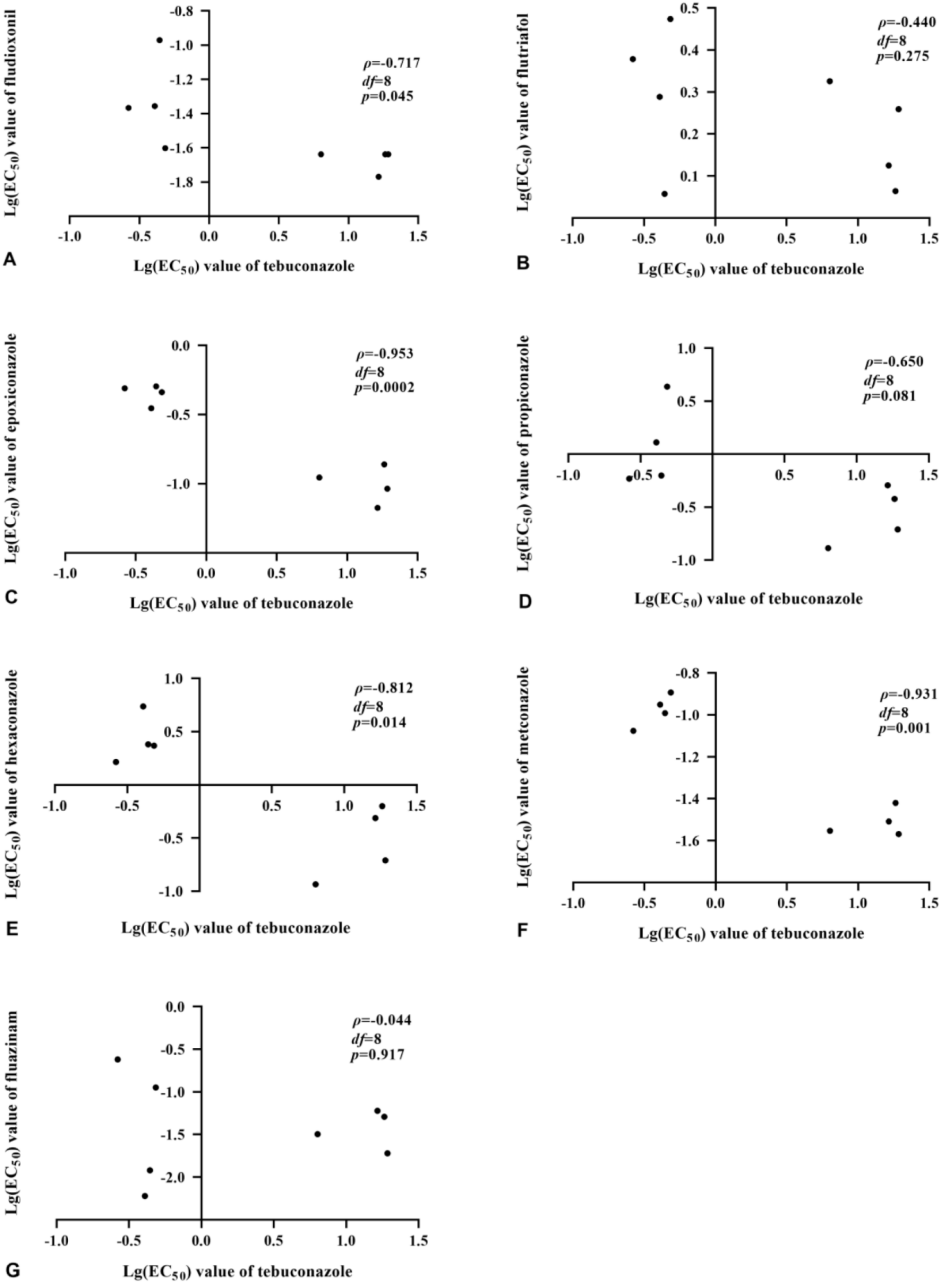
Cross-resistance between tebuconazole and seven alternative fungicides. Correlation analysis based on the log transformed EC_50_ values of each fungicide including **A**, fludioxonil; **B**, flutriafol; **C**, epoxiconazole; **D**, propiconazole; **E**, hexaconazole; **F**, metconazole; and **G**, fluazinam, which were derived from mycelial growth assays, in comparison to the equivalent values obtained for tebuconazole. Statistically significant correlations were ascribed according to the Spearman rank correlation coefficients (ρ > 0.8, *p* < 0.05)

Linear regression analysis (Fig. 2) indicated that there was significant negative cross-resistance between tebuconazole and the other fungicides assessed, which included fludioxonil, epoxiconazole, metconazole, and hexaconazole, with Spearman rank correlation coefficients (ρ > 0.8) of -0.717, -0.953, -0.812, and − 0.931, respectively. However, there was no similar evidence of cross-resistance between tebuconazole and the three fungicides with unrelated modes of action, including flutriafol, propiconazole, and fluazinam, which had Spearman coefficients of -0.440, -0.650, and − 0.044, respectively.

### Mycelial growth

**Fig. 3:**
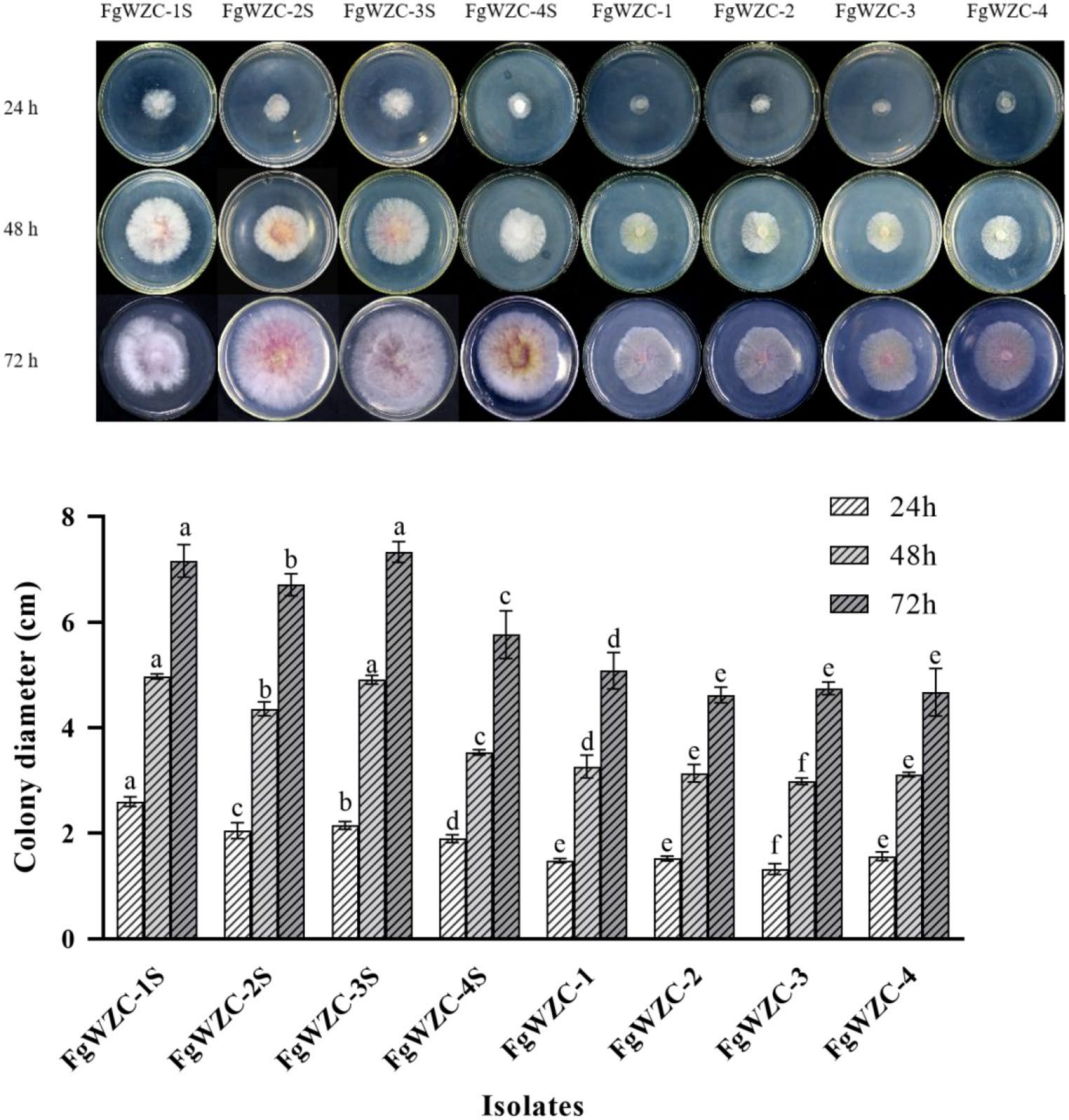
Mycelial growth of tebuconazole-resistant mutants of *F. graminearum. Note* Different letters indicate significant difference according to Fisher’s least significant difference test (*p* < 0.05). Same below

Mycelial growth assays revealed that the growth of the four tebuconazole-resistant mutants was significantly (*p* < 0.05) impaired compared to that of the wild-type parental isolates (Fig. 3), with the effect being noticeable to the naked eye even at the earliest time-point (24 h). The effect persisted throughout the entire period of observation (72 h), and occurred in all of the mutants assessed, which provided strong evidence of a fitness cost associated with tebuconazole resistance in *F. graminearum*.

### Sporulation and spore germination rate

**Fig. 4:**
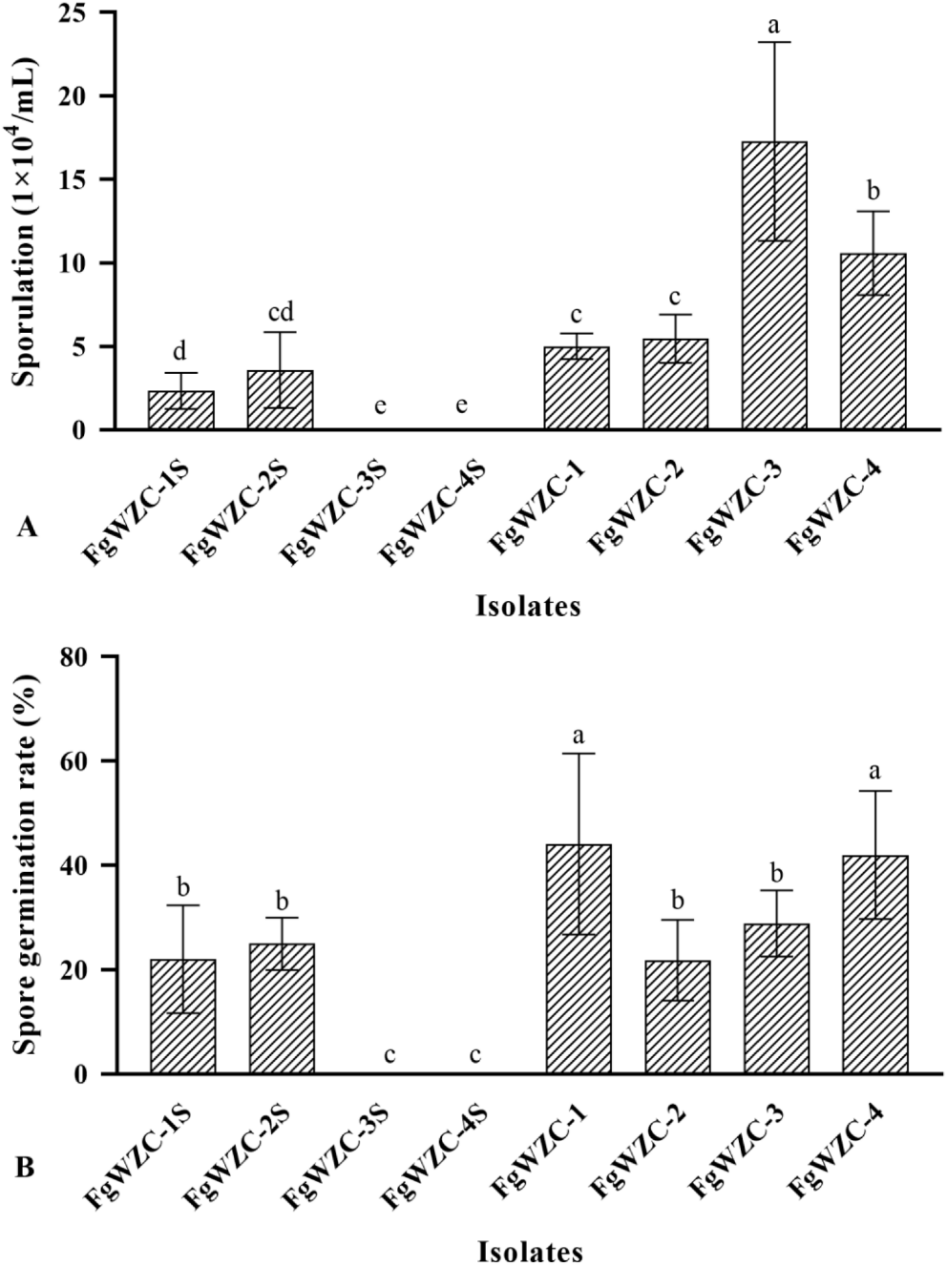
Sporulation and germination rates of tebuconazole-resistant mutants of *F. graminearum*. Note: (**A**) Sporulation. (**B**) Spore germination rate. Different letters indicate significant difference according to Fisher’s least significant difference test (*p* < 0.05)

The sporulation experiments indicated that the tebuconazole mutants produced a greater number of spores than their parental isolates (Fig. 4), which was particularly noteworthy with regard to FgWZC-3, and FgWZC-4, as their parental isolates FgWZC-3 S, and FgWZC-4 S, completely failed to sporulate under the experimental conditions implemented. The experiment also found evidence that tebuconazole-resistance might be associated with increased rates of spore germination, as one of the mutants (FgWZC-1) exhibited a significantly higher rate of germination compared to its parental isolate (FgWZC-1 S). However, the germination rate of a second mutant (FgWZC-2) exhibited no significant difference compared to its parental isolate (FgWZC-2 S), and no comparison could be made with the two non-sporulating isolates (FgWZC-3 S and FgWZC-4 S). Taken together, these results indicate a certain increase in fitness with regard to sporulation and spore germination, which might allow tebuconazole-resistant mutants to disperse more effectively under field conditions.

### Pathogenicity

**Fig. 5:**
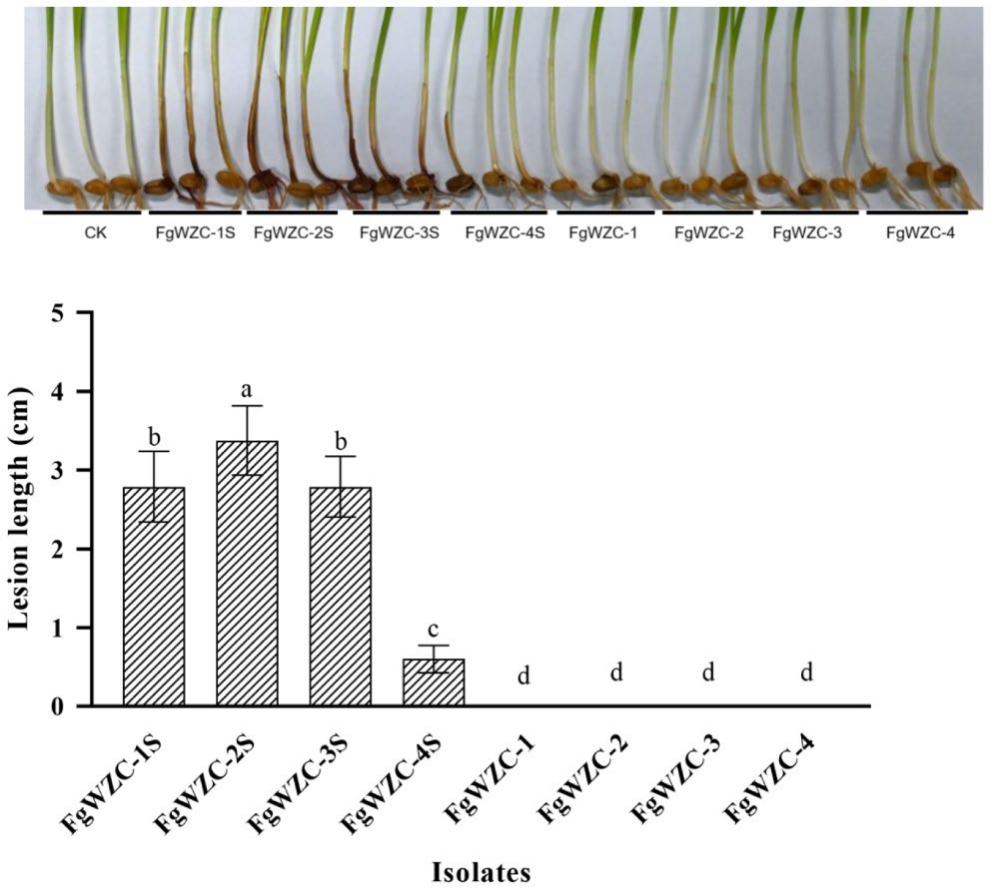
Pathogenicity of tebuconazole-resistant mutants of *F. graminearum*

Although all of the wild-type parental isolates were able to successfully infect wheat coleoptiles producing dark brown lesions that ranged in length from 0.5 to 4 cm, none of the tebuconazole mutants were able to form similar infections (Fig. 5). Indeed, the wheat coleoptiles inoculated with the 4 resistant mutants appeared no different to those treated with sterile water in the negative control. These results indicate a severe fitness cost associated with tebuconazole resistance causing a complete loss of pathogenicity even in wounded tissue, which indicates that such mutants would have severely impaired survivorship under field conditions.

### Stress response of four tebuconazole-resistant mutants of *F. graminearum*

#### Response to temperature

**Fig. 6:**
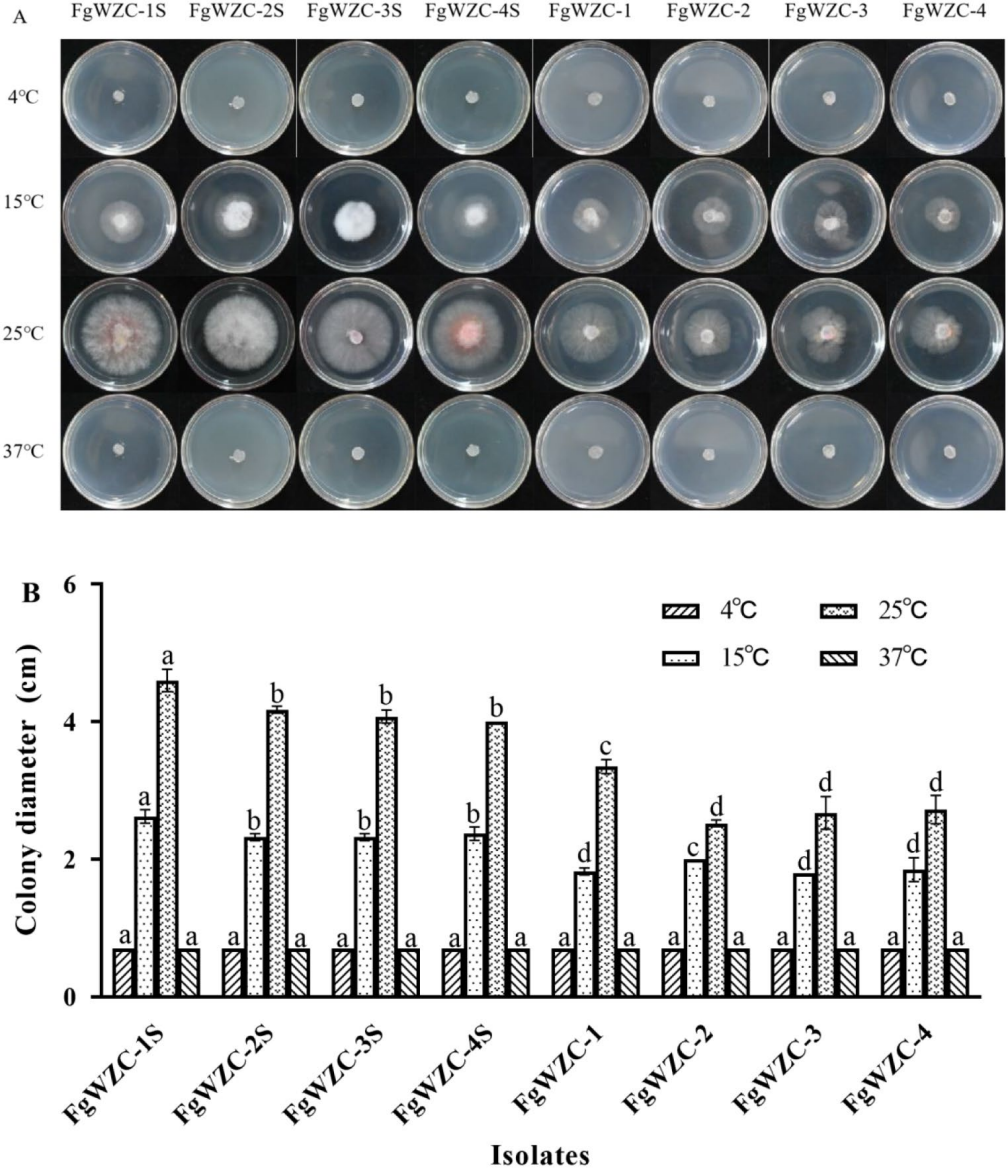
Effect of temperature on the growth of tebuconazole-resistant mutants of *F. graminearum*. Note: (**A**) Colony morphology at different culture temperatures for each isolate. (**B**) Colony diameter of the test strain. Different letters above the columns indicate significant difference according to Fisher’s least significant difference test (*p* < 0.05)

Mycelial growth assays revealed little difference between the response of the tebuconazole-resistant mutants and their wild-type parental isolates to temperature (Fig. 6), as neither were capable of growth at extremely low temperature (4 °C), or extremely high temperature (37 °C). However, as noted previously (Mycelial growth), at moderate temperatures similar to those encountered in the natural environment (15 °C and 25 °C), the tebuconazole-resistant mutants exhibited significantly (*p* < 0.05) reduced growth compared to their parental isolates, although the effect was slightly less discernable at lower temperature, when even the parental isolates exhibited reduced growth.

### Response to increased osmotic pressure

**Fig. 7:**
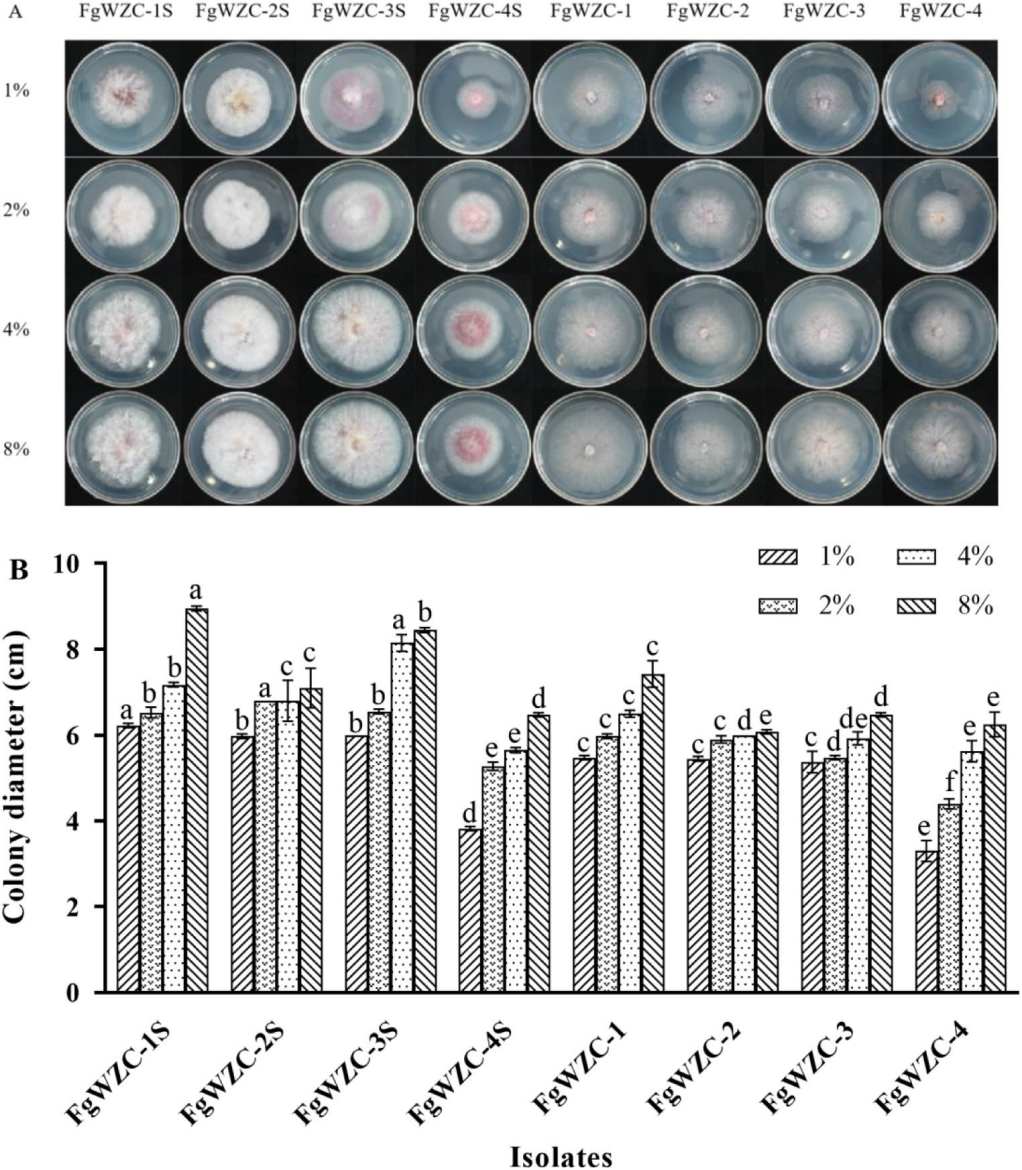
Effect of osmotic pressure on the growth of tebuconazole-resistant mutants of *F. graminearum*. Note: (**A**) Colony morphology of each isolate at different glucose concentrations. (**B**) Colony diameter of the test strain. Different letters above the columns indicate significant difference according to Fisher’s least significant difference test (*p* < 0.05)

The mycelial growth of both the tebuconazole-resistant mutants and the wild-type parental isolates was found to increase with the increased glucose concentration (1-8%), which was used as a proxy for increased osmotic pressure (Fig. 7). However, the tebuconazole-resistant mutants exhibited consistently reduced growth compared to their parental isolates, which although significant (*p* < 0.05) in every case, was not particularly great in nominal terms.

### Response to ionic stress

**Fig. 8:**
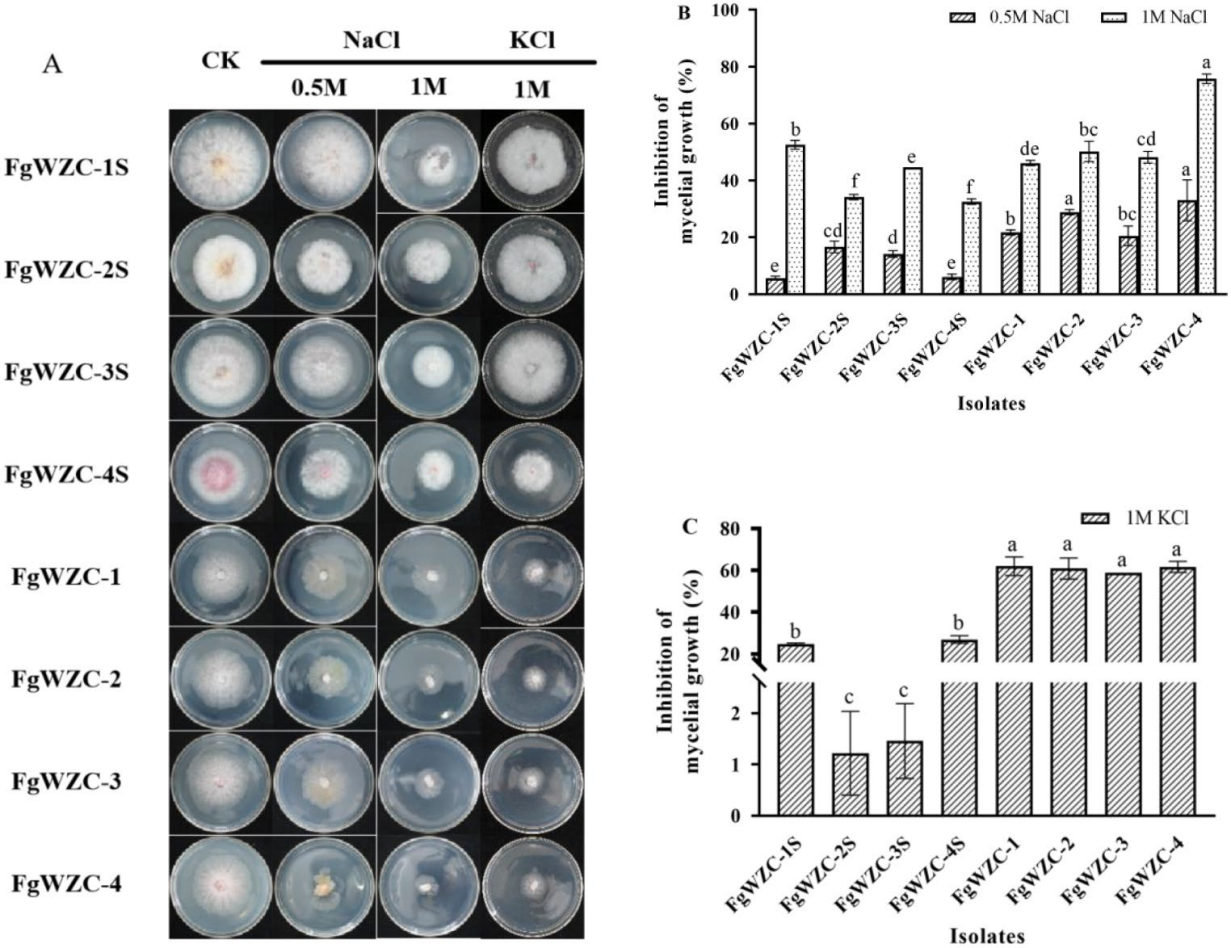
Effect of ionic stress on the growth of tebuconazole-resistant mutants of *F. graminearum*. Note: (**A**) Colony morphology of each isolate at different ion concentrations. (**B**) 0.5 M and 1 M NaCl. (**C**) 1 M KCl. Different letters above the columns indicate significant difference according to Fisher’s least significant difference test (*p* < 0.05)

Ionic stress was found to have a significant (*p* < 0.05) effect on the mycelial growth of both the tebuconazole-resistant mutants as well as their wild-type parental isolates (Fig. 8). For example, the addition of 0.5 M Na^+^ reduced growth by up to 20% in the parental isolates, and 30% in the resistant mutants (Fig. 8A and B). This inhibitory effect increased to as much as 50% and 80%, respectively, when the concentration was raised to 1 M Na^+^. A similar pattern of growth inhibition was also observed in response to 1 M K^+^. However, in this case the difference between the parental isolates (15–25% inhibition) and the tebuconazole-resistant mutants (65% inhibition) was much greater.

### Response to Congo Red, SDS and oxidative stress

**Fig. 9:**
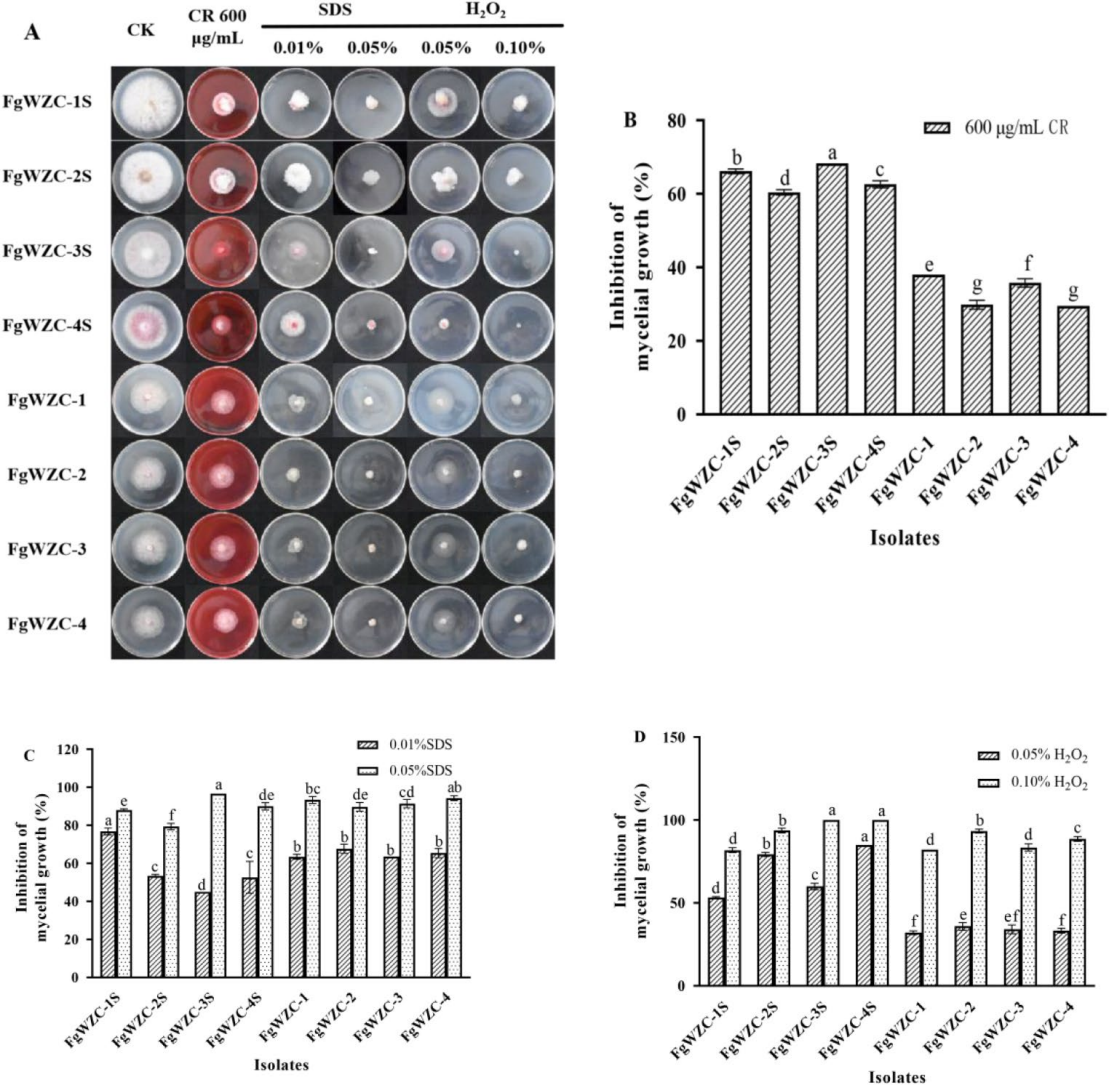
Effects of Congo red, SDS, and oxidative stress on the growth of tebuconazole-resistant mutants of *F. graminearum*. Note: (**A**) Colony morphology of each isolate growing for 7 days at 25 °C on PDA plates modified by various osmotic stress factors. (**B**) Congo red (600 µg/mL CR). (**C**) SDS (0.01% and 0.05%). (**D**) Oxidative stress (H_2_O_2_: 0.05% and 0.10%). Different letters above the columns indicate significant difference according to Fisher’s least significant difference test (*p* < 0.05)

The tebuconazole-resistant mutants were found to differ from their parental isolates in their response to Congo red, SDS, and oxidative stress (Fig. 9). For example, it was found that although all the isolates tested were sensitive to Congo red, and exhibited reduced mycelial growth (Fig. 9A and B), the effect was significantly (*p* < 0.05) more pronounced in the parental isolates (60-65% inhibition) compared to the resistant mutants (30-35% inhibition). The resistant mutants also differed in their response to SDS, although this was only noticeable at lower concentrations (0.01%), since at higher concentrations (0.05%) there was almost a complete loss of growth in all of the test isolates (Fig. 9A and C). However, this effect was not uniform, as one mutant (FgWZC-1) appeared less sensitive to 0.01% SDS, while the other three were more sensitive. Furthermore, although the differences were significant (*p* < 0.05), they were relatively small in nominal terms. Similarly oxidative stress had a dramatic effect on all of the test isolates, particularly at higher H_2_O_2_ concentration (0.1%), when growth was reduced by as much as 80-100% (Fig. 9A and D). As with the Congo red treatment, the resistant mutants appeared to be less sensitive compared to their parental isolates, which was more noticeable at the lower concentration (0.05% H_2_O_2_), when the parental isolates exhibited a significantly (*p* < 0.05) greater reduction in growth (50-80% inhibition), compared to the resistant isolates (25-30% inhibition).

Taken together, these results indicate that the stress responses of the tebuconazole-resistant mutant differed quite dramatically in comparison to those of the parental isolates. In general, the resistant mutants appeared more sensitive to stress, as was the case for osmotic and ionic stress, but in some instance they were less sensitive, for example in response to Congo red or oxidative stress.

### Sequence analysis of three *CYP51* genes from tebuconazole-resistant *F. graminearum* mutants

Comparisons of the DNA sequencing and predicted amino acid sequences obtained from the tebuconazole-resistant mutants and their wild-type parental isolates identified several point mutations that resulted in amino acid changes in the predicted *CYP51* sequences of the mutants (Table 2; Supplementary Fig. 1; Supplementary Fig. 2). For example, the *FgCYP51A* subunit of FgWZC-2 was found to contain two substitutions (I281T and T314A) (Table 2; Supplementary Fig. 1). In addition, FgWZC-2 was also found to have a further mutation in its *FgCYP51C* sequence (S195F) (Table 2; Supplementary Fig. 1). Similarly, mutations were also found in the *FgCYP51C* subunits of two other mutants, including five (Q332V, V333L, L334G, M399T, and E507G) in the sequence from FgWZC-3, and one (E267G) in the Fg*CYP51*C sequence of FgWZC-4 (Table 2; Supplementary Fig. 2). Interestingly, Interestingly, no amino acid changes were detected in the *FgCYP51B* sequences of any of the resistant mutants, and furthermore no point mutations were found in any of the sequences obtained from FgWZC-1, indicating that an alternative mechanism might be responsible for the observed resistance of this mutant.

**Table 2:** Amino acid changes in the predicted sequence of three FgCYP51 subunits from tebuconazole-resistant mutants of *F. graminearum*

Genes	Mutant	Nucleotide change	Amino acid change
*FgCYP51A*	FgWZC-1	/	/
	FgWZC-2	T591C, T920C, A1018G	I281T, T314A
	FgWZC-3	/	/
	FgWZC-4	/	/
*FgCYP51B*	FgWZC-1	A193G	/
	FgWZC-2	A337G, G905A, T1712C	/
	FgWZC-3	/	/
	FgWZC-4	/	/
*FgCYP51C*	FgWZC-1	T255C	/
	FgWZC-2	C639T	S195F
	FgWZC-3	T201C, G467A, C1049T, T1251C, T1414C, A1621G	Q332V, V333L, L334G, M399T, E507G
	FgWZC-4	A855G	E267G

*Note* “/” indicates that there is no valid data

### Relative expression of three *FgCYP51* genes in tebuconazole-resistant mutants of *F. Graminearum*

**Fig. 10:**
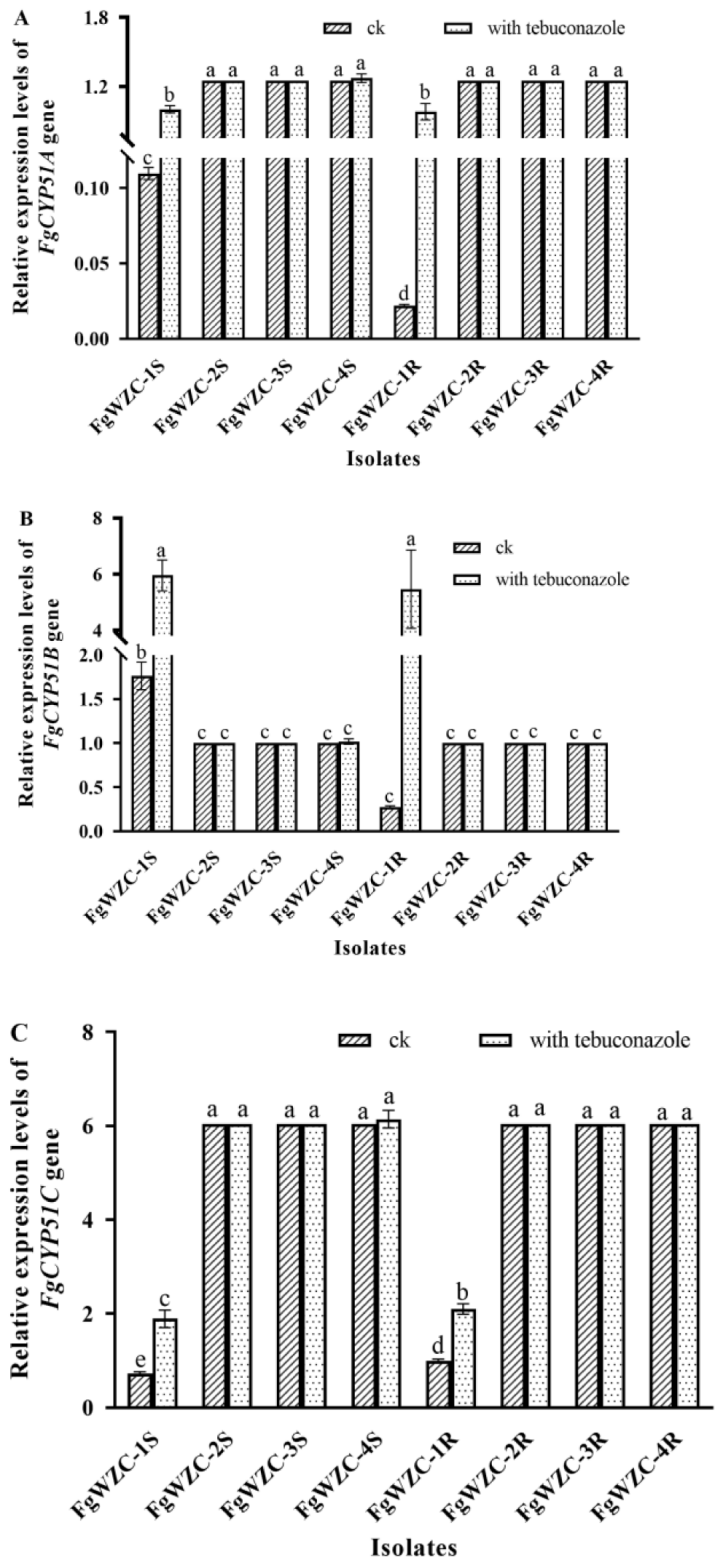
Relative expression of three FgCYP51 genes in tebuconazole-resistant mutants of *F. graminearum*. Note: (**A**) Relative expression of three FgCYP51A genes. (**B**) Relative expression of three FgCYP51B genes. (**C**) Relative expression of three FgCYP51C genes. Different letters above the columns indicate significant difference according to Fisher’s least significant difference test (*p* < 0.05)

The patterns of gene expression revealed by the qPCR analysis demonstrated a high degree of uniformity with regard to both the tebuconazole-resistant mutants and the parental isolates, as well as for the three different *CYP51* genes (Fig. 10). For example, three of the mutants (FgWZC-2, FgWZC-3, and FgWZC-4) exhibited an identical level of expression of their three *CYP51* genes, whether in the absence, or presence of the fungicide (0.1 µg/mL), although each gene varied relative to the actin reference gene, with *FgCYP51A* being 1.2 fold higher (Fig. 10A), *FgCYP51B* 1-fold higher (Fig. 10B), and *FgCYP51C* 6-fold higher (Fig. 10C). Interestingly the three wild-type parental isolates (FgWZC-2 S, FgWZC-3 S, and FgWZC-4 S) also exhibited this identical pattern of expression. The one exception to this trend was FgWZC-1, and its parental isolate FgWZC-1 S. In this case, the expression of each of the three genes was found to exhibit up-regulation in response to the presence of the fungicide. In the case of *FgCYP51A* and *FgCYP51B*, the basal level of expression in the mutant (FgWZC-1), was significantly (*p* < 0.05) lower than its parental isolate (FgWZC-1 S). However, in the presence of the fungicide, expression was significantly (*p* < 0.05) up-regulated in both the mutant and the parental isolate, which then had similarly elevated levels of expression (Fig. 10A and B). A less dynamic pattern of expression was observed for *FgCYP51C* in which the presence of the fungicide caused a doubling of expression in both the mutant (FgWZC-1) and parental isolate (FgWZC-1 S), with the mutant having significant (*p* < 0.05), but only slightly higher level of expression in both cases. It was also interesting to note that the expression of *FgCYP51A* and *FgCYP51C* in FgWZC-1 and FgWZC-1 S was never as high as that observed in the other test isolates (Fig. 10A and C), but that their expression of *FgCYP51B* could far exceed that of the other isolates in the presence of tebuconazole (Fig. 10B). It was also interesting to note that FgWZC-1 was the only mutant to completely lack any amino acid changes in its *CYP51* sequences, which could indicate that its divergent patterns of expression might in some way be linked to its observed tebuconazole resistance.

## Discussion

The *Fusarium* head blight caused by *F. graminearum* is one of the most destructive diseases affecting wheat production. Although it has a global distribution, it is a disease that is strongly influenced by climate, frequently causing epidemics in warm and humid conditions. In addition to causing serious loss to yield, *F. graminearum* produces mycotoxins that pose a serious threat to both human and livestock health. Tebuconazole is a triazole fungicide with good protective and curative action against *Fusarium* head blight, and is considered an environmentally friendly option for disease control. Its primary mode of action targets the biosynthesis of ergosterol, resulting in damage to the fungal membranes and cell death [25]. Previous studies have shown that tebuconazole can effectively control *Fusarium* head blight in the field [26], not only increasing wheat yield, but also reducing contamination with mycotoxins [15]. However, a recent report found that 32.4% of the *F. graminearum* isolates collected from various locations in the Henan Province of China, exhibited resistance to tebuconazole, indicating that resistance was already widespread in this region [16]. The current study, evaluated the tebuconazole sensitivity of 165 *F. graminearum* isolates collected from the Huang-Huai-Hai wheat-producing region of China, which encompasses Henan and four other provinces, over a five year period from 1919 to 2023, and in contrast found little evidence of tebuconazole resistance. Mycelial growth assays found that the EC_50_ values of the isolates ranged from 0.005 to 2.029 µg/mL, with an average EC_50_ of 0.33 ± 0.03 µg/mL, while the frequency distribution was found to conform to a normal distribution, indicating not only that there was no widespread occurrence of tebuconazole resistance in Huang-Huai-Hai, but also that the average EC_50_ was a suitable baseline for monitoring the emergence of resistance in the field. Although, the EC_50_ values varied from year to year, there was no trend of reduced sensitivity that might indicate the emergence of tebuconazole resistance, and is possible that these differences resulted from the prevailing climatic conditions, or the fungicide regiment implemented by farmers in particular years. However, one isolate with an exceptionally high EC_50_ of 2.029 was recovered from 2022, which could indicate that the development of field resistance was in its earliest stages.

The relative fitness of fungicide-resistant pathogens is an important consideration when assessing the risk of resistance emerging to different antifungal compounds. The current study found that although four tebuconazole mutants produced under laboratory conditions exhibited the potential for increased sporulation and spore germination rates, which might allow them to disperse more efficiently, they also exhibited significantly (*p* < 0.05) reduced mycelial growth, and completely lacked the ability to infect host tissue, which would severely limit their survivorship in the field. Similar results concerning reduce growth and pathogenicity were found in a study of tebuconazole resistance in *Rhizocotonia solani*, although in this case the mutants were also found to have increased tolerance of high osmotic pressure, which might indicate a reduced sensitivity to abiotic stress in general. However, the current study found that on the whole the four tebuconazole-resistant mutants of *F. graminearum* appeared to be more sensitive to adverse conditions, including osmotic and ionic stress, but that they were less sensitive to Congo red and oxidative stress.

Another important criterion when assessing the risk of fungicide resistance is the potential for cross-resistance with other commonly used fungicides. Although previous studies have shown that there can be cross-resistance between tebuconazole and other triazole fungicides [18], there is a large volume of evidence indicating that there is little cross-resistance with other fungicides that have unrelated modes of action such as prothionil, cypermethrin, isocarbamide, thiram, chlorothalonil, difenoconazole, pyrazole and carbendazim [20, 27–30]. These previous observations were confirmed by the current study, which found no evidence of negative cross-resistance with triazole fungicides including fludioxonil, epoxiconazole, metconazole, and hexaconazole. Such findings have implications for more rational fungicide use, where compounds with different modes of action can be used either in rotation, or in combination to prevent the emergence of resistance in field populations of *F. graminearum*, and thereby sustain profitable production in major wheat growing regions such as Huang-Huai-Hai.

Many previous studies have shown that mutations in the tebuconazole target protein, *CYP51*, can be linked to tebuconazole resistance in plant pathogenic fungi. For example, four amino acid substitutions, including S94A, N406S, L750P and H793R in the *CYP51* of *Rhizoctonia solani* have been associated with tebuconazole resistance [31], while the Y137H substitution was associated with resistance in *Villosiclava virens* [32]. It is interesting to note that an identical Y137H substitution in the *FgCYP51*B sequence has also been associated with tebuconazole resistance in *F. graminearum*, which has three different *CYP51* homologues. The current study found further evidence that amino acid changes might be associated with tebuconazole resistance, though all of them were different to those documented previously. For example, two substitutions (I281T, and T314A) were identified in the *FgCYP51A* subunit, while seven (S195F, Q332V, V333L, L334G, M399T, E507G, and E267G) occurred in the *FgCYP51*C subunit. Although further research is required to clarify the contribution such mutations might play in the tebuconazole resistance of *F. graminearum*, it is interesting to note that no conserved mutations were identified in the *FgCYP51* sequences, and that the substitutions most commonly occurred in the *FgCYP51*C subunit, which does not seem to play a role in ergosterol biosynthesis, but is required for full virulence [23]. It is also interesting to note that one mutant did not exhibit any amino acid changes in its *CYP51* sequences at all. However, it was found that this mutant did exhibit a dynamic pattern of expression, which resulted in dramatic up-regulation of all its *CYP51* genes in the presence of tebuconazole. Such altered expression has been observed in previous studies of *F. graminearum*, with Zhao et al. finding that altered *FgCYP51B* expression might play a role in resistance to the DMI fungicide prochloraz [22], and Zhang et al. noting that *FgCYP51A* was up-regulated in metconazole-resistant mutants [18]. Taken together, these observations indicate that tebuconazole resistance in *F. graminearum* is more complex than previously thought, and further research is required to completely characterize the various mechanisms by which it is achieved.

## Conclusions

In summary, the current study found that the *F. graminearum* population in Huang-Huai-Hai remains sensitive to tebuconazole, and established a baseline sensitivity for future monitoring. Investigation of laboratory mutants revealed that tebuconazole resistance was associated with reduced fitness, including a complete loss of pathogenicity. Molecular analysis revealed that altered expression and amino acid changes associated with the target protein *CYP51* could be linked to tebuconazole resistance, although more research is required to establish the precise mechanisms that might be responsible. However, the lack of cross-resistance between tebuconazole and unrelated fungicides, indicates that the risk of resistance can be reduced by rotation, or combined application of fungicides with different modes of action, and therefore that tebuconazole remains an extremely effective fungicide for control of the *Fusarium* head blight caused by *F. graminearum*, and sustaining profitable harvests in Huang-Huai-Hai, an important wheat producing region of China.

## Methods

### Experimental materials and fungicides

Infected wheat ear samples (provided by wheat engineering center of henan institute of science and technology) were collected in the Huang-Huai-Hai wheat producing region of China over 5 consecutive years beginning in 2019. After tissue isolation and single spore purification, a total of 165 individual isolates of *F. graminearum* were identified. Each isolate was subcultured on PDA before mycelial samples were suspended in 20% glycerol for long-term storage at 4 °C.

All of the test fungicides used in the current study, including 95.0% tebuconazole, 95.0% fludioxonil, 95.0% epoxiconazole, 95.0% metconazole, 96.5% fluazinam, 95.2% propiconazole, 97.0% flutriafol and 95.0% hexaconazole (Hubei Jianyuan Chemical Co., Ltd., Hubei, China), were dissolved in acetone to prepare 5.0 × 10^4^ µg/mL stock solutions, which were stored in the dark at 4°C until required.

### Establishment of a baseline tebuconazole sensitivity for *F. Graminearum* Mycelial growth assay

The mycelial growth assay described in a previous study [33] was used to determine the effect of tebuconazole treatment on *F. graminearum*. Briefly, each isolate was initially established on PDA by inoculation from the glycerol stock, and culture at 25 °C for 3 days. Mycelial plugs (7 mm) were then taken from the colony margin and transferred to fresh PDA plates amended with tebuconazole at the following concentrations: 0, 0.025, 0.05, 0.1, 0.2, 0.4, 0.8, 1.6, and 3.2 µg/mL. The resulting plates were then incubated at 25 °C for a further 3 days before the diameter of the colony was measured and used to construct the inhibition curve required to calculate the half maximal effective concentration (EC_50_) values as described previously [34].

### Frequency distribution curve and establishment of baseline sensitivity

The EC_50_ value of each isolate were used to construct the frequency distribution curve by sorting the data into five intervals as described in a previous study [35]. Starting from the minimum value, the number of corresponding strains was counted and its frequency calculated using the average EC_50_ value of all the *F. graminearum* isolates as the abscissa, and the isolate distribution frequency as the ordinate. The frequency distribution histogram was constructed using GraphPadPrism9 software, with the normal distribution corroborating the average EC_50_ as a suitable measure of baseline tebuconazole sensitivity.

### Changes in tebuconazole sensitivity over time

Potential changes in tebuconazole sensitivity were assessed by comparing the average EC_50_ values of the isolates collected in each of the successive years during the period of study.

### Biological characteristics of tebuconazole-resistant mutants of *F. Graminearum*

#### Preparation of resistant mutants

The sensitivity of wild-type strains collected was determined, and it was found that most strains were sensitive to tebuconazole, so four strains with high sensitivity were randomly selected for drug domestication to obtain tebuconazole-resistant Fusarium graminearum mutants. That is, highly sensitive isolates were selected using the data from the mycelial growth assays (FgWZC-1 S, FgWZC-2 S, FgWZC-3 S and FgWZC-4 S, which had EC_50_ values of 0.441, 0.407, 0.484 and 0.264 µg/mL, respectively), and tebuconazole-resistant mutants prepared by repeated exposure to fungicide under laboratory conditions [30, 34]. Each wild-type isolate was initially prepared by dark-culture on PDA at 25 °C for 4 days, before mycelial plugs (7 mm) were transferred to media containing 2, 6, 10–15 µg/mL tebuconazole by successive subculture at 25 °C for 4 days, each time transferring any surviving colonies to fresh media with an increased fungicide concentration. Repeated screening resulted in four tebuconazole resistant mutants corresponding to each of the parental isolates, namely: FgWZC-1, FgWZC-2, FgWZC-3 and FgWZC-4, which had EC_50_ values of 16.192, 19.281, 6.349 and 18.304 µg/mL, respectively.

#### Cross-resistance between tebuconazole and other fungicides

The mycelial growth assay described above was adapted to investigate the potential for cross-resistance between tebuconazole and other triazole fungicide including epoxiconazole, metconazole, propiconazole, and hexaconazole, as well as other fungicides with alternative modes of action such as fluazinam, flutriafol, and fludioxonil. Fungicide specific EC_50_ values were determine for both the tebuconazole-resistant mutants and their wild-type parental isolate using the fungicide concentrations listed in Table 3. Potential correlations were evaluated by plotting the log(EC_50_) of tebuconazole as the abscissa, and the log(EC_50_) of each other fungicide as ordinates as described in previous studies [28, 36], with Spearman rank analysis being used to assess the statistical confidence of any interactions (ρ > 0.8, *p* < 0.05).

**Table 3:** Concentration range of test fungicides

Fungicides	Concentration(µg/mL)
Tebuconazole^S^	0, 0.025, 0.05, 0.1, 0.2, 0.4, 0.8, 1.6, 3.2
Tebuconazole^R^	0, 0.8, 1.6, 3.2, 6.4, 12.8, 25.6, 51.2, 102.4
Propiconazole	0, 0.1, 0.2, 0.4, 0.8, 1.6, 3.2, 6.4, 12.8
Flutriafol	0, 0.1, 0.2, 0.4, 0.8, 1.6, 3.2, 6.4, 12.8
Fluazinam	0, 0.003125, 0.00625, 0.0125, 0.025, 0.05, 0.1, 0.2, 0.4
Epoxiconazole	0, 0.025, 0.05, 0.1, 0.2, 0.4, 0.8, 1.6, 3.2
Fludioxonil	0, 0.0125, 0.025, 0.05, 0.1, 0.2, 0.4, 0.8, 1.6
Metconazole	0, 0.003125, 0.00625, 0.0125, 0.025, 0.05, 0.1, 0.2, 0.4
Hexaconazole	0, 0.1, 0.2, 0.4, 0.8, 1.6, 3.2, 6.4, 12.8

*Note*^S^ represents the concentration used with the sensitive wild-type isolates, and ^R^ with the tebuconazole-resistant mutants

### Mycelial growth of tebuconazole-resistant mutants

#### The mycelial growth assay was also used to compare the fitness of the tebuconazole-resistant mutants with their sensitive parental isolates

Sporulation and spore germination rate of tebuconazole-resistant mutants.

Sporulation was induced using the mung bean method detailed in a previous study [37], in which 100 mL conical flasks containing 75 mL 60% mung bean broth were inoculated with 6 mycelial plugs (7 mm) and incubated for 36 h at 25 °C with shaking at 150 rpm. The spores were then collected by filtration and centrifugation (6000 rpm for 10 min), and the number of conidia produced evaluated by counting the number occurring in diluted samples using a hemocytometer. The germination rate was then assessed by diluting spore suspensions to a concentration of 1 × 10^− 4^ spores/mL with a 0.1% glucose solution, and counting the number of germinated spores after 6 h incubation at room temperature.

Pathogenicity of tebuconazole-resistant mutants.

The pathogenicity of the tebuconazole-resistant mutants was compared to that of their parental isolates using wheat coleoptiles as described previously [37]. Wheat grains were first surface sterilized by soaking them in 75% alcohol for 30 min, and washing them 3 times in sterile water. The prepared grains were then kept in a sealed container for 1.5 days at room temperature to allow them to germinate, at which point they were inoculated with 10 µL of a conidia suspension (1 × 10^− 4^ spores/mL), or sterile water as a negative control. The inoculated coleoptiles were incubated at room temperature for 14 days before the lesions were photographed and measured, with each being assigned to one of three categories: low pathogenicity (0 ~ 0.5 cm), moderate pathogenicity (0.5 ~ 1.0 cm), or high pathogenicity (> 1.0 cm). Each treatment consisted of ten coleoptiles with the entire experiment being repeated three times.

### Response of tebuconazole-resistant mutants of *F. Graminearum* to abiotic stress

#### Temperature stress

The mycelial growth assay described previously was used to investigate the effect of temperature on the tebuconazole-resistant mutants simply by incubating fresh PDA cultures at 4 °C, 15 °C, 25 °C and 37 °C for 4 days, and then measuring the diameter of the resulting colonies. Identical plates were inoculated with the sensitive parental isolates for comparison.

#### Osmotic stress

The response of the tebuconazole-resistant mutants to increasing osmotic pressure was assessed using the mycelial growth assay with PDA amended with 1%, 2%, 4% or 8% glucose. The colony diameter was measured after incubation at 25 °C for 4 days.

Ionic stress.

The response of the tebuconazole-resistant mutants to ionic stress was assessed using the mycelial growth assay in conjunction with PDA amended with either NaCl (0.5 M and 1 M), or KCl (1 M), with the colony diameter being measured after incubation at 25 °C for 4 days.

#### Congo Red, SDS and oxidative stress

The cell wall integrity of the tebuconazole-resistant mutants was assessed using Congo red, while their response to surfactant and oxidative stress were assessed by exposure to SDS and H_2_O_2_, respectively [38–40]. The mycelial growth assay described above was adapted using PDA amended with either Congo red (600 µg/mL), SDS (0.01% or 0.05%), or H_2_O_2_ (0.05% or 0.1%). The colony diameter was measured after incubation at 25 °C for 4 days.

#### Molecular analysis

Cloning and sequencing of the *FgCYP51A*, *FgCYP51B* and *FgCYP51C* genes.

Fresh mycelium samples were collected from each of the *F. graminearum* mutant and wild-type isolates, and total DNA extracted using the CTAB method [35]. Primers (Table 4) designed to the full-length sequences of the three *FgCYP51* genes (*FgCYP51A*, FGSG_04092; *FgCYP51B*, FGSG_01000; and *FgCYP51C*, FGSG_11024) obtained from the EnsemblFungi database (http://fungi.ensembl.org/index.html) were synthesized by the Shanghai Biological Engineering Co., Ltd (Shanghai, China), and used to amplify the three *CYP51* genes from the different DNA samples in 20 µL reaction mixtures containing 0.1 µg template DNA, 0.25 µmol/L of each primer, 10 µL 2×Taq PCR StarMix, and 8 µL sterile distilled water (Table 5). The PCR itself was performed using the thermocycler and the following program: initial denaturation at 94 °C for 2 min, followed by 35 cycles of denaturation at 98 °C for 30 s, annealing at 53 °C for 30 s (*FgCYP51B* at 65 °C), and extension at 72 °C for 45 s, followed by a final extension at 72 °C for 8 min. The resulting PCR products were purified, cloned into the pMDTM19-T vector, and sequenced commercially (Wuhan JinKaiRui Biological Engineering Co., Ltd, Wuhan, China).

**Table 4:** Primers used in the current study

Primers	Sequence (5’-3’)	Purpose
FgCYP51A-F	ATGTTCCATCTACTCATCTATC	Amplification of full-length *FgCYP51A* gene
FgCYP51A-R	CTATATCTTCTTCCTACGCTCC	
FgCYP51B-F	ATGGGTCTCCTTCAAGAAC	Amplification of full-length *FgCYP51B* gene
FgCYP51B-R	TTACTGGCGTCGCTCCCAGTG	
FgCYP51C-F	ATGGAATCGCTCTACGAGAC	Amplification of full-length *FgCYP51C* gene
FgCYP51C-R	TCATTCTACTGTCTCGCGTC	
Rt -Fg-actin-F	GTCCACCTTCCAGCAAATGT	Amplification of the actin reference gene
Rt -Fg-actin-R	CCCAAAGCTTAGCGTCTGTC	
RT-FgCYP51A-F	AGCCCGTACTTGCCCTTTGG	Expression analysis of *FgCYP51A* gene
RT-FgCYP51A-R	GGGCGGGTCGTGAGAACAAA	
RT-FgCYP51B-F	GAGTCCCTGGCCGCTCTCTA	Expression analysis of *FgCYP51B* gene
RT-FgCYP51B-R	GCGGCGCTCCTTGATAGTGT	
RT-FgCYP51C-F	TTCGTCTCCCGGCACAATGG	Expression analysis of *FgCYP51C* gene
RT-FgCYP51C-R	CGTCCAGCTCCAAAGGGCAA	

**Table 5:** Composition of PCR reaction mix

Component	Concentration (µL)
DNA Template	1.0
Upstream Primer	0.5
Downstream Primer	0.5
2×Taq PCR StarMix (Dye)	10.0
Sterile Water	8.0

### Relative expression of three Fg*CYP51* genes in tebuconazole-resistant mutants of *F. Graminearum*

The relative expression of the three *FgCYP51* genes (*FgCYP51A*: 1574 bp; *FgCYP51B*: 1749 bp; *FgCYP51C*: 1655 bp) was assessed in both the tebuconazole-resistant mutants and the wild-type parental isolates, in either the absence or presence of tebuconazole (0.5 µg/mL) using the method of a previous study [41]. Total RNA was first extracted from fresh mycelium samples using a fungal RNA kit (Omegabio-tek, Basel, Switzerland), before the cDNA was synthesized using the PrimeScriptRT kit (TaKaRa, Kusatsu, Japan). Gene-specific primer sets including RT-*FgCYP51A*-F/RT-*FgCYP51A*-R, RT-Fg*CYP51*B-F/RT-Fg*CYP51*B-R and RT-Fg*CYP51*C-F/RT-Fg*CYP51*C-R (Table 2) were then used to amplify partial sequences of the *FgCYP51A*, *FgCYP51B*, and *FgCYP51C* genes, respectively, using the system and the fluorescent dye (TaKaRa, Japan), with actin as the reference gene. The qPCR itself was processed using the thermocycler (ThermoFisher, Waltham, MA, USA) with the following program: an initial denaturation at 95 °C for 10 s, followed by 40 cycles of 95 °C for 5 s, 60 °C for 32 s, and dissociation at 95 °C for 15 s, at 60 °C for 60 s, and 95 °C for 15 s.

### Data analysis

The statistical analysis of the biological characteristics of tebuconazole-resistant mutants of *F. graminearum* data collect in the study was performed using the Spss26 software package, while the EC_50_ frequency distribution and cross-resistance data were plotted using GraphPadPrism9 software.

## Electronic supplementary material

Below is the link to the electronic supplementary material.

Supplementary Material 1

Supplementary Material 2

## Data Availability

All data generated or analyzed in this study are available from the corresponding author on reasonable request. The data during the current study are available from the NCBI Nucleotide database under accession numbers CM000575, CM000574 and CM000576.
