# Supplementary material for: Baseline tebuconazole sensitivity and potential resistant risk in Fusarium graminearum
Source: BMC Plant Biol. 2024 Aug 21;24:789. doi: 10.1186/s12870-024-05206-1 (PMC11337888; doi:10.1186/s12870-024-05206-1)
Supplement: Supplementary file 1 — Supplementary Material 1 [file 12870_2024_5206_MOESM1_ESM.docx]

Supplementary Figures


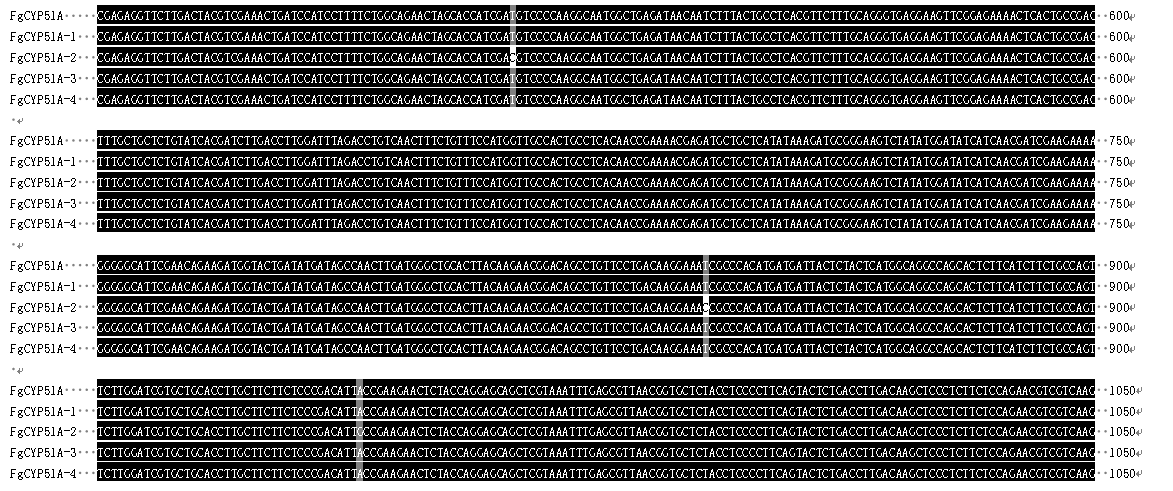

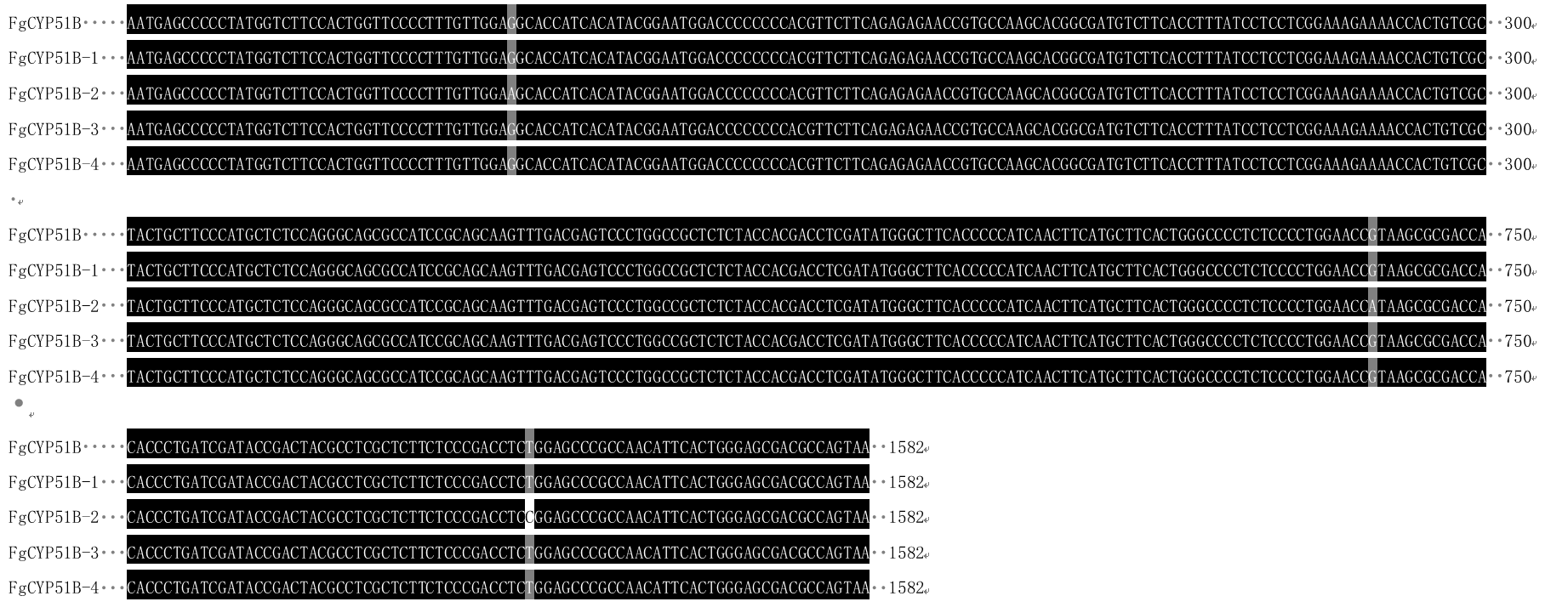

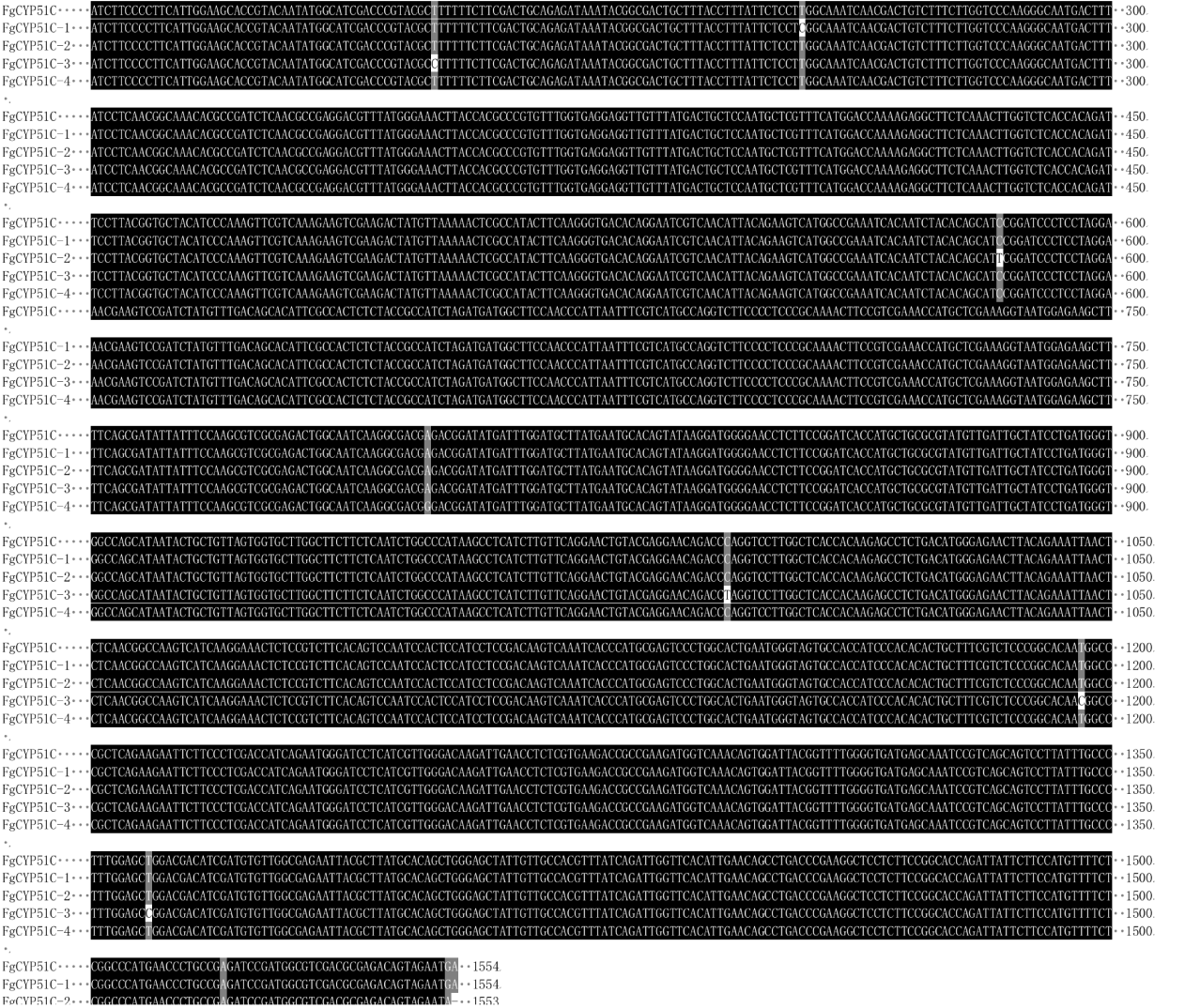


Supplementary Fig. 1 Base sequence alignment results of 4 tebuconazole-resistant mutants


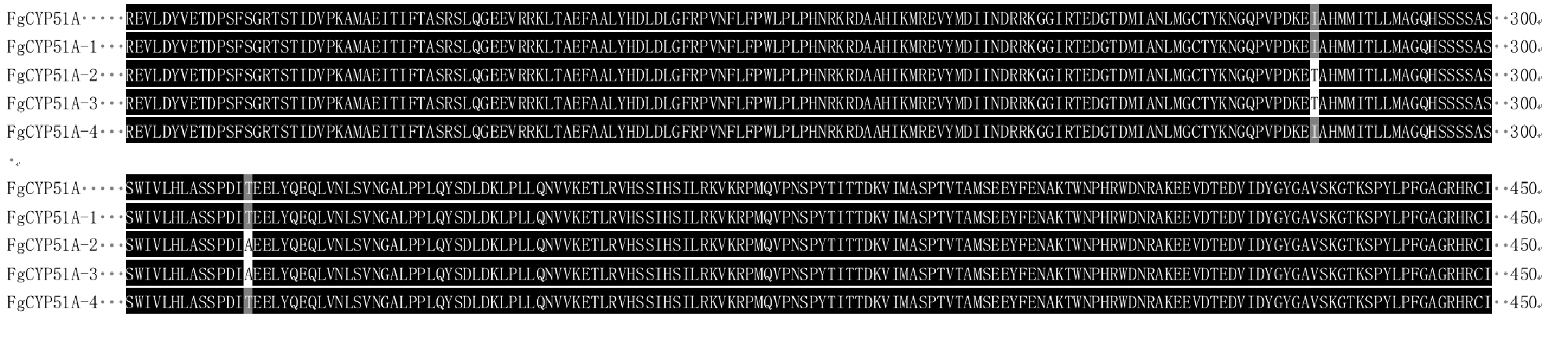

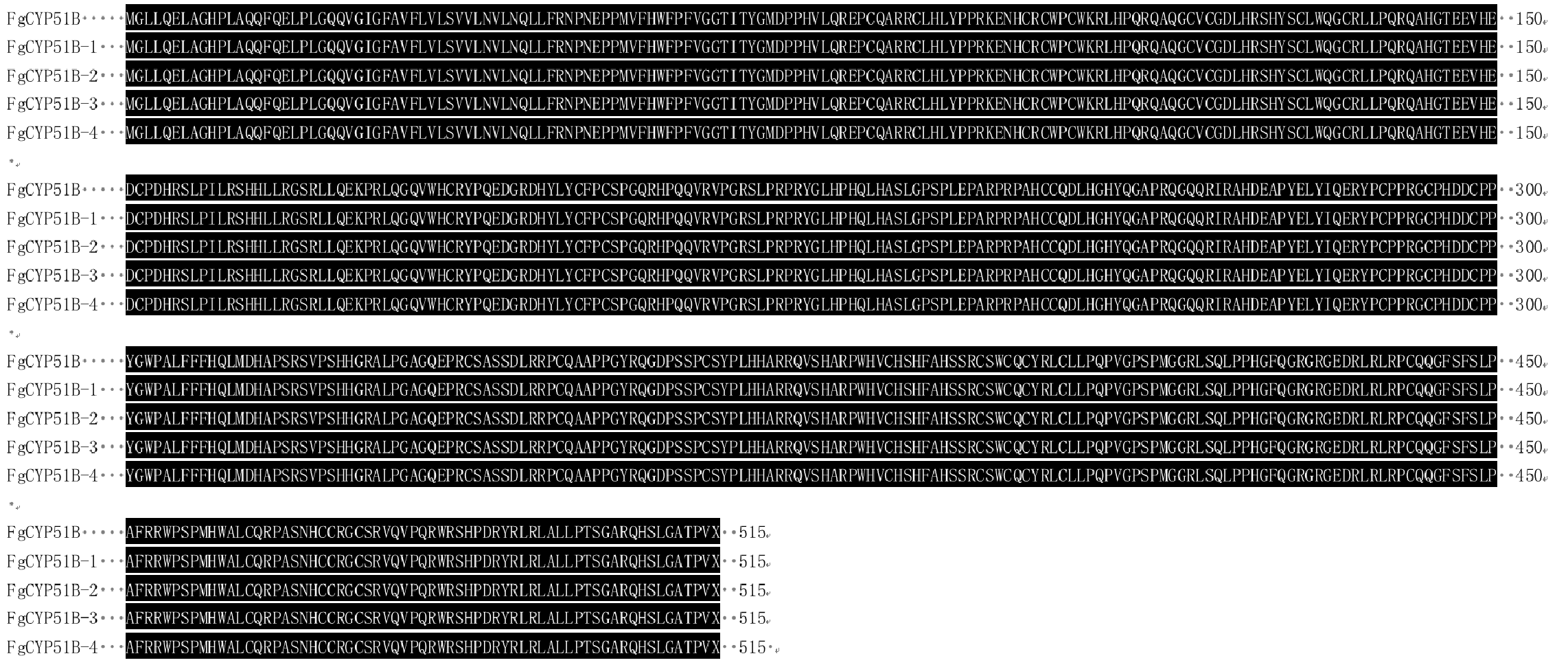

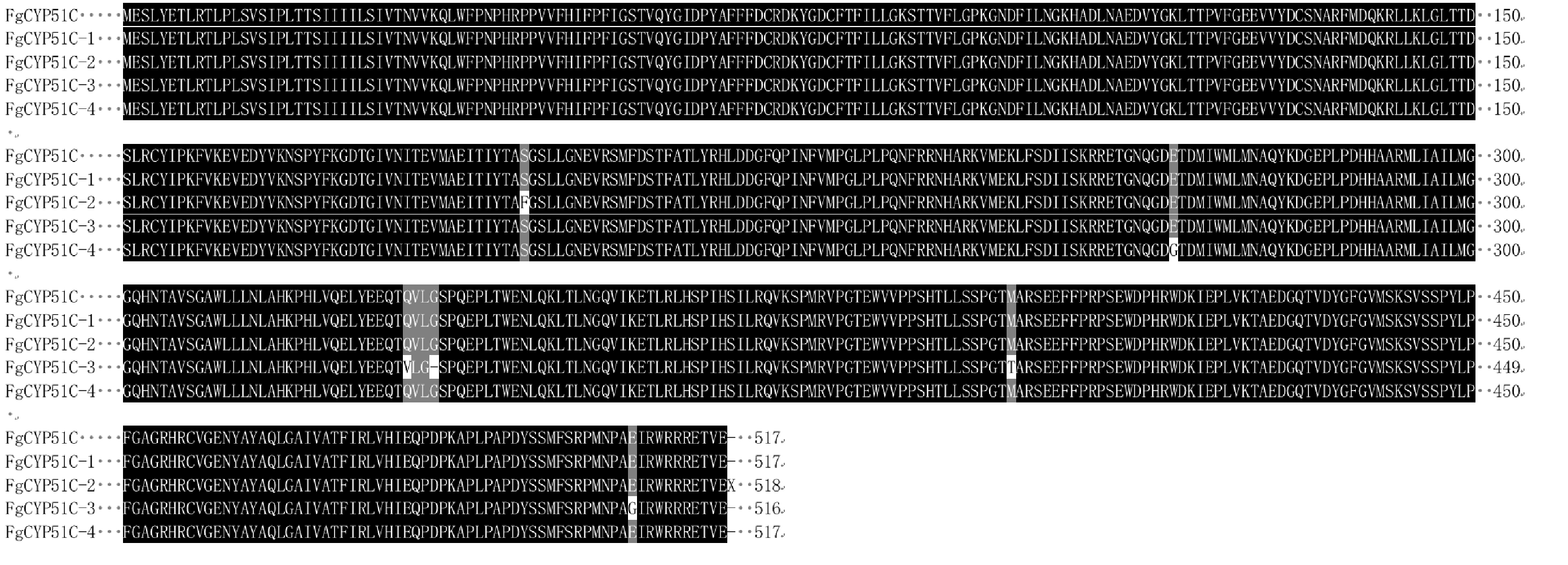


Supplementary Fig. 2 Mino acid sequence alignment results of 4 tebuconazole-resistant mutants
