# Supplementary material for: Baseline tebuconazole sensitivity and potential resistant risk in Fusarium graminearum
Source: BMC Plant Biol. 2024 Aug 21;24:789. doi: 10.1186/s12870-024-05206-1 (PMC11337888; doi:10.1186/s12870-024-05206-1)
Supplement: Supplementary file 2 — Supplementary Material 2 [file 12870_2024_5206_MOESM2_ESM.docx]

Supplementary Table

Supplementary Table 1 Tebuconazole sensitivity (EC_50_) of 165 *F. graminearum* isolates collected from wheat felds in the Huang-Huai-Hai, China between 2019 and 2023

| Isolates | Years | EC_50_ (μg/mL) | Isolates | Years | EC_50_ (μg/mL) |
| --- | --- | --- | --- | --- | --- |
| XMCM2019-SZ-1-1 | 2019 | 0.401 | XMCM-2022-DB-2 | 2022 | 0.169 |
| XMCM2019-SZ-1-2 | 2019 | 0.368 | XMCM-2022-DB-3 | 2022 | 0.107 |
| XMCM2019-SZ-1-3 | 2019 | 0.333 | XMCM-2022-DB-4 | 2022 | 0.985 |
| XMCM2019-SZ-2-1 | 2019 | 0.221 | XMCM-2022-DB-5 | 2022 | 0.576 |
| XMCM2019-SZ-2-2 | 2019 | 0.257 | XMCM-2022-DB-6 | 2022 | 0.238 |
| XMCM2019-SZ-3-1 | 2019 | 0.110 | XMCM-2022-DB-7 | 2022 | 0.500 |
| XMCM2019-SZ-3-2 | 2019 | 0.155 | XMCM-2022-DB-8 | 2022 | 0.794 |
| XMCM2019-SZ-4-1 | 2019 | 0.098 | XMCM2022-ZK-1-1 | 2022 | 0.791 |
| XMCM2019-SZ-4-2 | 2019 | 0.399 | XMCM2022-ZK-1-2 | 2022 | 1.828 |
| XMCM2019-SZ-5-1 | 2019 | 0.239 | XMCM2022-ZK-2-1 | 2022 | 1.586 |
| XMCM2019-SZ-6-1 | 2019 | 0.044 | XMCM2022-ZK-2-2 | 2022 | 1.455 |
| XMCM2019-SZ-7-1 | 2019 | 0.363 | XMCM2022-ZK-2-3 | 2022 | 1.230 |
| XMCM2019-SZ-7-2 | 2019 | 0.274 | XMCM2022-ZK-3-1 | 2022 | 0.715 |
| XMCM2019-SZ-7-3 | 2019 | 0.157 | XMCM2022-ZK-3-2 | 2022 | 0.619 |
| XMCM2019-SZ-8-1 | 2019 | 0.481 | XMCM2022-ZK-3-3 | 2022 | 0.950 |
| XMCM2019-SZ-8-2 | 2019 | 0.444 | XMCM2022-ZK-4-1 | 2022 | 1.590 |
| XMCM2019-SZ-9-1 | 2019 | 0.135 | XMCM2022-ZK-5-1 | 2022 | 0.300 |
| XMCM2019-HA-1-1 | 2019 | 0.452 | XMCM2022-ZK-5-2 | 2022 | 0.783 |
| XMCM2019-HA-1-2 | 2019 | 0.391 | XMCM2022-ZK-5-3 | 2022 | 0.286 |
| XMCM2019-HA-2-1 | 2019 | 0.425 | XMCM2022-ZK-5-4 | 2022 | 1.579 |
| XMCM2019-HA-2-2 | 2019 | 0.184 | XMCM2022-ZK-6-1 | 2022 | 0.705 |
| XMCM2019-HA-3-1 | 2019 | 0.227 | XMCM2022-ZK-6-2 | 2022 | 2.029 |
| XMCM2019-HA-4-1 | 2019 | 0.367 | XMCM2022-ZK-7-1 | 2022 | 0.595 |
| XMCM2019-HA-4-2 | 2019 | 0.273 | XMCM2022-ZK-7-2 | 2022 | 0.341 |
| XMCM2019-HA-5-1 | 2019 | 0.346 | XMCM2022-ZK-8-1 | 2022 | 1.062 |
| XMCM2019-HA-5-2 | 2019 | 0.252 | XMCM2022-ZK-8-2 | 2022 | 0.720 |
| XMCM2019-HA-6-1 | 2019 | 0.248 | XMCM2022-ZK-9-1 | 2022 | 0.982 |
| XMCM2019-NJ-1-1 | 2019 | 0.183 | XMCM2022-ZK-9-2 | 2022 | 1.050 |
| XMCM2019-NJ-1-2 | 2019 | 0.277 | XMCM2022-ZK-9-3 | 2022 | 1.314 |
| XMCM2019-NJ2-1 | 2019 | 0.315 | XMCM2022-ZK-10-1 | 2022 | 0.025 |
| XMCM2019-NJ-2-2 | 2019 | 0.214 | XMCM2022-ZK-10-2 | 2022 | 0.029 |
| XMCM2019-SC-1-1 | 2019 | 0.229 | XMCM2022-ZK-10-3 | 2022 | 0.021 |
| XMCM2019-SC1-2 | 2019 | 0.296 | XMCM2022-ZK-10-4 | 2022 | 0.042 |
| XMCM2019-GY1-1 | 2019 | 0.424 | XMCM2022-ZK-10-5 | 2022 | 0.035 |
| XMCM2019-GY1-2 | 2019 | 0.349 | XMCM2022-ZK-11-1 | 2022 | 0.051 |
| XMCM2019-GZ-1-1 | 2019 | 0.437 | XMCM2022-ZK-11-2 | 2022 | 0.036 |
| XMCM2019-HN-1-1 | 2019 | 0.070 | XMCM2022-ZK-12-1 | 2022 | 0.051 |
| XMCM2019-XZ-1-1 | 2019 | 0.281 | XMCM2022-ZK-12-2 | 2022 | 0.046 |
| XMCM2020-SC-31 | 2020 | 0.172 | XMCM2023-HB-132 | 2023 | 0.292 |
| XMCM2020-SC-32 | 2020 | 0.127 | XMCM2023-HB-1 | 2023 | 0.158 |
| XMCM2020-GS-21 | 2020 | 0.288 | XMCM2023-HB-2 | 2023 | 0.190 |
| XMCM2020-GS-22 | 2020 | 0.333 | XMCM2023-HB-3 | 2023 | 0.158 |
| XMCM2020-NJ-17 | 2020 | 0.030 | XMCM2023-XC-5 | 2023 | 0.718 |
| XMCM2020-NJ-18 | 2020 | 0.032 | XMCM2023-XC-6 | 2023 | 0.604 |
| XMCM2020-HA-29 | 2020 | 0.027 | XMCM2023-XC-7 | 2023 | 0.876 |
| XMCM2020-HA-30 | 2020 | 0.006 | XMCM2023-XC-31 | 2023 | 0.224 |
| XMCM2020-HN-23 | 2020 | 0.029 | XMCM2023-XC-35 | 2023 | 0.549 |
| XMCM2020-HN-24 | 2020 | 0.005 | XMCM2023-XC-41 | 2023 | 0.406 |
| XMCM2020-XZ-19 | 2020 | 0.010 | XMCM2023-XC-42 | 2023 | 0.416 |
| XMCM2020-XZ-20 | 2020 | 0.010 | XMCM2023-XC-54 | 2023 | 0.151 |
| XMCM2020-XZ-25 | 2020 | 0.012 | XMCM2023-XC-57 | 2023 | 0.326 |
| XMCM2020-XZ-126 | 2020 | 0.011 | XMCM2023-XC-28 | 2023 | 0.492 |
| XMCM2020-SZ-27 | 2020 | 0.010 | XMCM2023-XC-56 | 2023 | 0.452 |
| XMCM2020-SZ-28 | 2020 | 0.010 | XMCM2023-XC-60 | 2023 | 0.477 |
| XMCM2020-HB-12 | 2020 | 0.016 | XMCM2023-XC-64 | 2023 | 0.371 |
| XMCM2020-HB-13 | 2020 | 0.018 | XMCM2023-XC-65 | 2023 | 0.375 |
| XMCM2020-HB-14 | 2020 | 0.017 | XMCM2023-AY-93 | 2023 | 0.416 |
| XMCM2020-HB-15 | 2020 | 0.024 | XMCM2023-AY-105 | 2023 | 0.389 |
| XMCM2020-HB-16 | 2020 | 0.011 | XMCM2023-AY-107 | 2023 | 0.630 |
| XMCM2020-HB-1 | 2020 | 0.318 | XMCM2023-AY-108 | 2023 | 0.394 |
| XMCM2020-HB-2 | 2020 | 0.266 | XMCM2023-AY-110 | 2023 | 0.306 |
| XMCM2020-HB-3 | 2020 | 0.405 | XMCM2023-KQ-12 | 2023 | 0.142 |
| XMCM2020-HB-4 | 2020 | 0.213 | XMCM2023-KQ-82 | 2023 | 0.172 |
| XMCM2020-HB-5 | 2020 | 0.144 | XMCM2023-KQ-58 | 2023 | 0.232 |
| XMCM2020-HB-6 | 2020 | 0.103 | XMCM2023-KQ-63 | 2023 | 0.166 |
| XMCM2020-HB-7 | 2020 | 0.024 | XMCM2023-KQ-66 | 2023 | 0.059 |
| XMCM2020-HB-8 | 2020 | 0.027 | XMCM2023-KQ-17 | 2023 | 0.142 |
| XMCM2020-HB-9 | 2020 | 0.028 | XMCM2023-FQ-81 | 2023 | 0.143 |
| XMCM2020-HB-10 | 2020 | 0.034 | XMCM2023-FQ-17 | 2023 | 0.129 |
| XMCM2020-HB-11 | 2020 | 0.757 | XMCM2023-FQ-63 | 2023 | 0.124 |
| YMCM-2021-XX-42 | 2021 | 0.031 | XMCM2023-FQ-104 | 2023 | 0.152 |
| YMCM-2021-XX-44 | 2021 | 0.028 | XMCM2023-JZ-56 | 2023 | 0.137 |
| YMCM-2021-XX-46 | 2021 | 0.015 | XMCM2023-JZ-61 | 2023 | 0.172 |
| YMCM-2021-XX-48 | 2021 | 0.014 | XMCM2023-HB-122 | 2023 | 0.250 |
| YMCM-2021-XX-50 | 2021 | 0.018 | XMCM2023-HB-166 | 2023 | 0.782 |
| YMCM-2021-XX-51 | 2021 | 0.014 | XMCM2023-HB-117 | 2023 | 0.216 |
| YMCM-2021-XX-52 | 2021 | 0.016 | XMCM2023-HB-109 | 2023 | 0.103 |
| YMCM-2021-XX-53 | 2021 | 0.028 | XMCM2023-HB-102 | 2023 | 0.257 |
| YMCM-2021-XX-54 | 2021 | 0.017 | XMCM2023-HB-129 | 2023 | 0.088 |
| YMCM-2021-XX-12 | 2021 | 0.022 | XMCM2023-HB-138 | 2023 | 0.114 |
| YMCM-2021-XX-14 | 2021 | 0.013 | XMCM2023-HB-103 | 2023 | 0.323 |
| XMCM-2022-DB-1 | 2022 | 0.049 | XMCM2023-HB-126 | 2023 | 0.357 |
| XMCM2022-ZK-12-3 | 2022 | 0.038 | / | / | / |

Note: “/” indicates that there is no valid data.
